# Utilizing Wearable Device Data for Syndromic Surveillance: A Fever Detection Approach

**DOI:** 10.3390/s24061818

**Published:** 2024-03-12

**Authors:** Patrick Kasl, Lauryn Keeler Bruce, Wendy Hartogensis, Subhasis Dasgupta, Leena S. Pandya, Stephan Dilchert, Frederick M. Hecht, Amarnath Gupta, Ilkay Altintas, Ashley E. Mason, Benjamin L. Smarr

**Affiliations:** 1 Shu Chien-Gene Lay Department of Bioengineering, University of California San Diego, San Diego, CA 92093-0021, USA; bsmarr@ucsd.edu; 2 UC San Diego Health Department of Biomedical Informatics, University of California San Diego, San Diego, CA 92093-0021, USA; lbruce@ucsd.edu; 3 UCSF Osher Center for Integrative Health, University of California San Francisco, San Francisco, CA 92093-0021, USA; wendy.hartogensis@ucsf.edu (W.H.); leena.pandya@ucsf.edu (L.S.P.); rick.hecht@ucsf.edu (F.M.H.); ashley.mason@ucsf.edu (A.E.M.); 4 San Diego Supercomputer Center, University of California San Diego, San Diego, CA 92093-0021, USA; sudasgupta@ucsd.edu (S.D.); a1gupta@ucsd.edu (A.G.); ialtintas@ucsd.edu (I.A.); 5 Department of Management, Zicklin School of Business, Baruch College, The City University of New York, New York, NY 10010, USA; stephan.dilchert@baruch.cuny.edu; 6 Halıcıoğlu Data Science Institute, University of California San Diego, San Diego, CA 92093-0021, USA

**Keywords:** wearables, syndromic surveillance, illness detection

## Abstract

Commercially available wearable devices (wearables) show promise for continuous physiological monitoring. Previous works have demonstrated that wearables can be used to detect the onset of acute infectious diseases, particularly those characterized by fever. We aimed to evaluate whether these devices could be used for the more general task of syndromic surveillance. We obtained wearable device data (Oura Ring) from 63,153 participants. We constructed a dataset using participants’ wearable device data and participants’ responses to daily online questionnaires. We included days from the participants if they (1) completed the questionnaire, (2) reported not experiencing fever and reported a self-collected body temperature below 38 °C (negative class), or reported experiencing fever and reported a self-collected body temperature at or above 38 °C (positive class), and (3) wore the wearable device the nights before and after that day. We used wearable device data (i.e., skin temperature, heart rate, and sleep) from the nights before and after participants’ fever day to train a tree-based classifier to detect self-reported fevers. We evaluated the performance of our model using a five-fold cross-validation scheme. Sixteen thousand, seven hundred, and ninety-four participants provided at least one valid ground truth day; there were a total of 724 fever days (positive class examples) from 463 participants and 342,430 non-fever days (negative class examples) from 16,687 participants. Our model exhibited an area under the receiver operating characteristic curve (AUROC) of 0.85 and an average precision (AP) of 0.25. At a sensitivity of 0.50, our calibrated model had a false positive rate of 0.8%. Our results suggest that it might be possible to leverage data from these devices at a public health level for live fever surveillance. Implementing these models could increase our ability to detect disease prevalence and spread in real-time during infectious disease outbreaks.

## 1. Introduction

Public health agencies commonly use syndromic surveillance (SS) to augment a variety of traditional disease surveillance systems [1,2]. SS systems generally do not assess laboratory-confirmed reports and instead rely on the presence of detectable symptoms; cases are typically reported before the results of a laboratory test are available [1]. SS systems require a lower implementation burden relative to traditional surveillance systems that rely on case reports, such as the National Notifiable Disease Surveillance System. SS systems are, therefore, potentially (1) more scalable, (2) more sensitive, and (3) better able to more rapidly identify outbreaks [3,4]. Systems using commercially available wearable devices (wearables) to detect illness states exhibit many of the same strengths as SS. That is, they are (1) scalable, as in 2019, approximately 30% of US consumers already used wearables, which are relatively inexpensive [5]; (2) sensitive as wearable device physiological data can be monitored in large, distributed, diverse populations, and can be used to discern periods of relative health versus illness; and (3) rapid as wearable device data can be analyzed in near real-time.

Many recent efforts propose machine learning classifiers for the within-individual detection of specific, acute illnesses using wearable device data [6,7,8,9,10,11,12,13,14,15]. Other works have investigated using wearables to monitor population-level changes corresponding to influenza-like illnesses (ILI) [16,17]. Both within-individual detection and population-level monitoring tasks are tractable because wearables measure physiological metrics that are anomalous around acute illness onset. These anomalies can include increased heart rate (HR), respiratory rate (RR), and temperature, and decreased heart rate variability (HRV) and physical activity [13]. However, real-time SS systems hold the potential to detect such aberrations that may signal the increased prevalence of a novel pathogen [2]. As such, we sought to determine whether wearable device data could be used for generalized SS, and we evaluated such feasibility by focusing on fever detection.

Fever is often a crucial component of the case definition for many SS systems across conditions, including ILI, where the presence of fever is necessary but not sufficient for a case to be considered an ILI event [18]. Moreover, fever is sometimes the only symptom surveilled [19,20,21]. In this work, we explored changes in wearable-measured physiology around the onset of self-reported fevers, proposed a classifier for detecting its onset, and demonstrated the classifier’s performance in a broad population.

## 2. Materials and Methods

We previously reported on data collected for these analyses by Mason et al. [6]. Additional details on the recruitment and exclusion criteria of the initial cohort are outlined in Mason et al.; however, we outline details relevant to the subset of participants used in these analyses. The original cohort comprised 63,153 participants spanning 106 countries [22] who completed online questionnaires and wore the Oura Ring Gen2, a commercially available wearable device (Oura Health, Oulu, Finland) on a finger of their choosing. Participants completed baseline, monthly, and daily online questionnaires; the daily questionnaire included a checklist to report the subjective experience of a number of symptoms. These analyses focused on self-reported fever symptoms; participants could self-report the symptom “Fever” since they last completed a daily questionnaire (“*Have you experienced any of the following symptoms since you last did this survey? (Please check all that apply.)*”). Participants were also asked to self-report the highest body temperature reading they had taken during the last day by thermometry (*“If you took your temperature in the last day, what was the highest reading?”*).

To select days that were more likely to be from a fever event, we considered any day where a participant reported both (1) experiencing a self-reported fever and (2) a self-reported temperature greater than or equal to 38 °C to be a fever day. Fever days with wearable device data from at least seven nights over a fourteen-day baseline period and the nights before and after the fever day were included in the dataset. Wearable device data from the nights before and after fever days comprised positive class examples in the training set and the test set. Negative class examples comprised days wherein participants both (1) self-reported not experiencing fever and (2) self-reported a temperature lower than 38 °C (non-fever days). Non-fever days also had retrievable wearable device data from at least seven nights over a fourteen-day baseline period and the nights before and after the non-fever day.

Participants wore the Oura Ring Gen2 (Oura Health Oy, Oulu, Finland). The Oura Ring connects to the Oura App (available from the Google Play Store and the Apple App Store) via Bluetooth. Users can wear the ring continuously in both wet and dry conditions. The Oura Ring generates physiological metrics by aggregating data gathered from on-device sensors. These high-resolution metrics are transformed into summary metrics before their transmission to a smartphone app. The Oura Ring Gen2 uses a proprietary algorithm to estimate when a user is at rest and when they have gone to bed. After the Oura Ring determines that a user has gone to bed, the Oura Ring gathers a high-frequency photoplethysmogram (PPG), which it uses to calculate interbeat intervals (IBI), which are used in heart rate (HR), heart rate variability (HRV), and respiratory rate (RR). Both HR and HRV measured by Oura have been externally validated to be highly accurate [23]. RR has been validated internally by Oura and is claimed to be highly accurate compared to a medical-grade ECG, with a mean error of 0.71 breaths per minute and a correlation of 0.96 [24]. The Oura Ring Gen2 assesses a user’s dermal (distal) temperature throughout the day (i.e., not only when the user is in bed) using a negative temperature coefficient (NTC) thermistor on the internal surface of the ring. The NTC thermistor has been internally validated by Oura and has been shown to provide near-perfect agreement with a research-grade sensor [25]. During sleep, the Oura Ring uses a proprietary algorithm to estimate the stage of sleep a user is currently in. Sleep stages can be one of the following: awake, REM, light (N1 or N2), or deep (N3). This algorithm has been externally validated and is 79% accurate for four-stage sleep stage classification [26]. Further details regarding these sensors and the algorithms used to determine HR, HRV, RR, and sleep stages are provided in Appendix A. High-resolution metrics are transformed into summary metrics before transmission to a smartphone app. Oura further aggregates these summary metrics across each period of detected sleep into a “sleep summary”. The dataset used in these analyses comprises metrics (“sleep summary metrics”) from the longest sleep of the day (i.e., the sleep summary with the greatest total time spent asleep). We included all sleep summary metrics generated by Oura that were single, scalar, and physiologically interpretable values. Sleep summaries also included metrics that we did not include, i.e., arrays of HR and HRV across every 5 min of sleep, strings that specify the start and end of detected bedtimes, or any of the metrics that are a proprietary combination of the metrics we included (i.e., so-called “sleep scores”). Table 1 lists each sleep summary metric included in these analyses, along with detailed descriptions.

The input features to our model follow the standard format for a binary classification task. Let D = {(x_1_, y_1_)… (x_n_, y_n_)} be the training dataset. x_j_ ∈ R^k^ and y_j_ ∈ {0, 1}. x_j_ is a vector of size k = 35. Entries {1,…, 14} in x_j_ $\overset{\mathrm{def}}{=}$ z_i,m_ are as follows:

$$z_{i,m}=\frac{{Night}_{i,m}-\mu_{\left(-14\to -28\right),m}}{\sigma_{\left(-14\to -28\right),m}}$$

**Figure 1:**
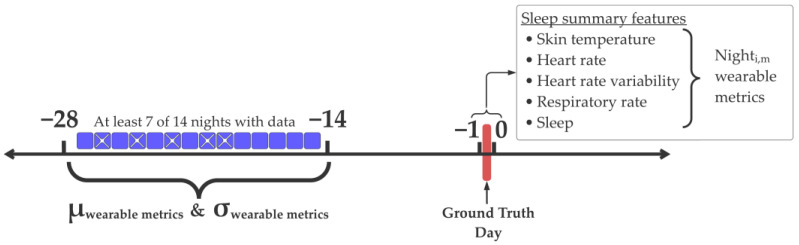
Instance selection and normalization procedure. At least 7 out of the 14 days in the range of −28 to −14 relative to the ground truth day were retrievable. The mean (μ) and standard deviation (σ) from these days were used to normalize z-score wearable device metrics. We depict an example of a valid instance with its baseline period (−28 → −14) with retrievable data from 9 out of 14 nights (nights without retrievable data are indicated by a white cross). This instance is based on sleep summary features from the night before (night −1) and the night after (night 0) relative to the ground truth day.

Here, the z-scored wearable device metrics from the night before (Night −1, Figure 1) are from the ground truth day. Similarly, entries {15,…, 28} in x_j_ $\overset{\mathrm{def}}{=}$ z_i,m_ are from the night after (Night 0, Figure 1) the ground truth day. Entries {29,…, 35} in x_j_ $\overset{\mathrm{def}}{=}$ ∈ {0, 1} correspond to one-hot-encoded Boolean features for the day of the week (Sunday through Monday) of the ground truth day. In summary, the features are (1) z-scored sleep summary metrics (x_i,m_) from the night before (NB) and the night after (NA) each fever or non-fever day and (2) one-hot-encoded Boolean features for the day of the week (Sunday through Monday) of the ground truth day. We included the day of the week as a feature, given the tendency for human weekly rhythms (i.e., alcohol consumption [7]) to drive physiological changes that manifest similarly to acute illnesses. It is y_j_ = 0 if the jth example is from a non-fever day and y_j_ = 1 if the jth example is from a fever day. A schematic describing the normalization procedure and instance selection process is shown in Figure 1.

In order to ensure applicability, we implemented a relatively simple, commonly used ensemble classifier based on the standard implementation of a Histogram-Based Gradient-Boosting Classification Tree from the sklearn Python (Open source) package v1.2.0 (sklearn.ensemble.HistGradientBoostingClassifier) with all hyper-parameters left at default. Models of this variety are commonly used for physiological anomaly detection [8,10,27,28]. For training and testing, we followed a five-fold stratified cross-validation scheme with a user split as previously outlined in Merill et al. [15], where each model was trained on data from a subset of participants and tested on another subset. We stratified users based on whether that user had a fever day.

Classifiers could be calibrated during training, which aligns a classifier’s predicted class probabilities and the empirical likelihood of events occurring [29]. Predictions from well-calibrated classifiers tend to more accurately reflect real-world outcomes. Importantly, this can allow practitioners to choose intervention thresholds based on a classifier’s predictions, which can lead to more precise resource allocation and risk assessment [30]. We used logistic (sigmoid) regression with a two-fold split to calibrate our model using the sklearn v1.2.0 implementation (sklearn.calibration.CalibratedClassifierCV). We used the Brier score to assess the extent to which our classifier was calibrated [31]. The Brier score was calculated by taking the squared difference between the classifier’s predicted probability and the corresponding outcome (0 for incorrect predictions and 1 for correct ones). The Brier score was then the mean squared difference across all predictions. Brier scores ranging from 0 to 1 and lower values indicate a more calibrated classifier. We used the sklearn v1.2.0 implementation of the Brier score (sklearn.metrics.brier_score_loss).

We examined the relative importance of each wearable and measured physiological change in our classifier using permutation importance, which is a data-driven approach that quantifies the weight that a tree-based classifier places on individual features [32]. Permutation importance is determined by evaluating how much a classifier’s performance degrades after the systematic perturbation of a specific feature. Baseline classification performance is established on the unperturbed dataset. Then, each individual feature (i.e., the z-score and average HR from the night before a [non]-fever day) is randomly permuted between examples (i.e., all [non]-fever days) in the dataset. This permutation disrupts any relationship between the feature and the classification output. The change in classification performance is determined after permutation. Features, when permuted, that cause the largest drop in classification performance are the most important. We used the sklearn v1.2.0 permutation importance (sklearn.inspection.permutation_importance) with 30 permutations per feature at each iteration of the five cross-validation.

The receiver operating characteristic (ROC) and Precision–Recall curves are often used to visually assess binary classification performance [33]. The ROC illustrates the relationship between a classifier’s true positive rate (i.e., recall, sensitivity) and false positive rate (i.e., 1-specificity) across predicted probability threshold values. The ROC curve is often used to examine the trade-off between correctly identifying positive instances and incorrectly classifying negative instances as positive. The integration of the ROC yields the area under the ROC (AUROC), which is commonly used to summarize the ROC. On the other hand, the Precision–Recall curve (PRC) plots precision (i.e., positive predictive value) against recall (i.e., true positive rate, sensitivity) across predicted probability threshold values. The PRC can more accurately represent the performance on imbalanced datasets; this method describes a classifier’s ability to correctly identify positive examples while minimizing false positives. Average (i.e., mean) precision (AP) is frequently used to summarize the PRC.

## 3. Results

**Figure 2:**
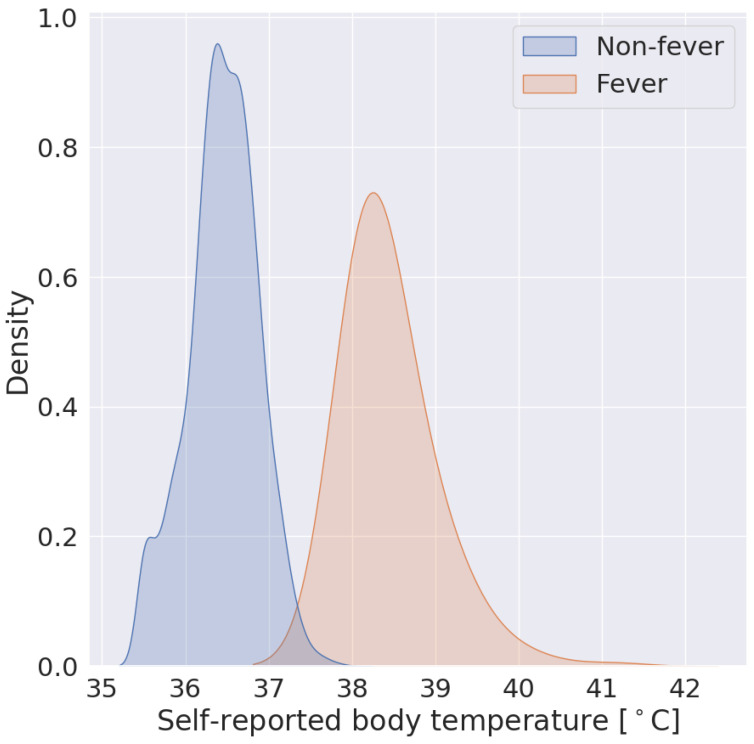
Self-reported body temperatures from non-fever examples are in blue and fever examples are in orange.

Sixteen thousand, seven hundred, and ninety-four participants provided at least one valid ground truth day; there were a total of 724 fever days (positive class examples) from 463 participants and 342,430 non-fever days (negative class examples) from 16,687 participants. The mean self-reported body temperature was 38.45 (SD = 0.50) for fever days and 36.45 (SD = 0.42) for non-fever days. The distributions of self-reported body temperatures can be found in Figure 2. Table 2 provides the characteristics of participants included in these analyses. The average participant age was 47.2 years; 43.6% were women.

**Figure 3:**
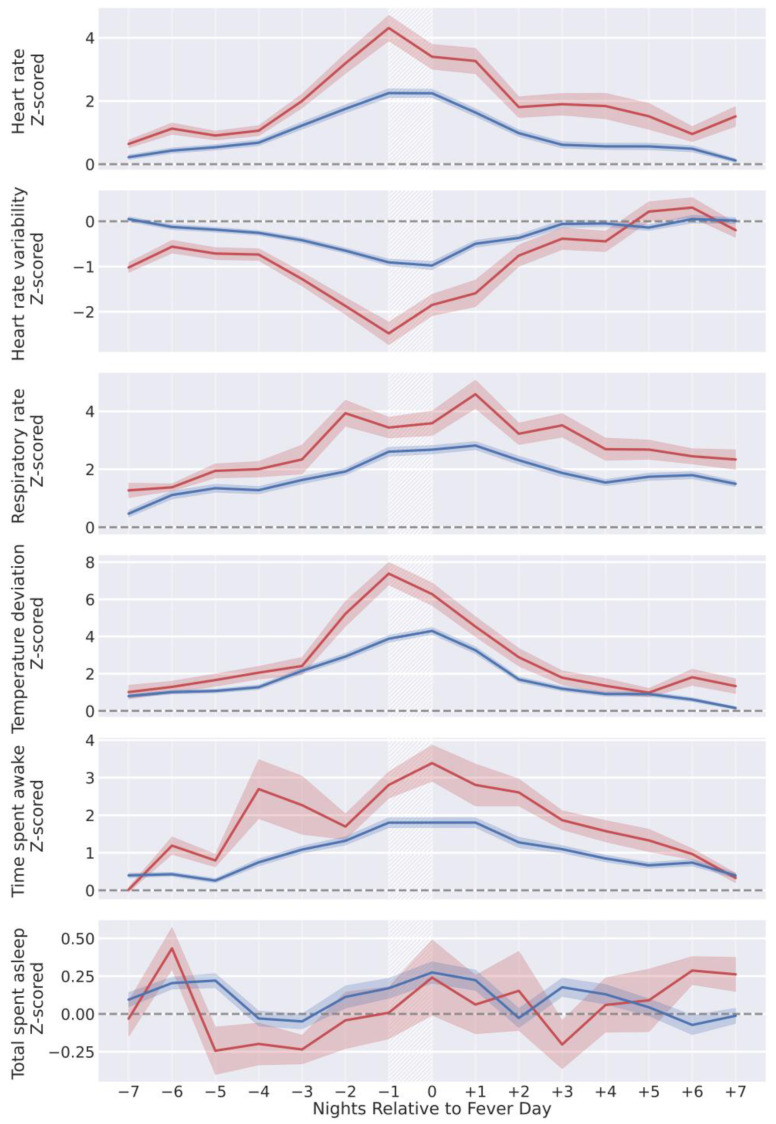
Z-score-normalized wearable metrics from individuals, aligned by self-reported fever day (white hatched areas) and grouped by self-reported temperature on fever day. Individuals reporting temperatures in the range of (38–39 °C) are in blue (*n* = 621), and (39+ °C) are in red (*n* = 103). Lines represent the mean z-score normalized wearable metric across all participants in the respective group for each night, and shaded regions are the 95% confidence interval of the mean.

Wearable-measured physiological changes in the nights before and after fever days appear in Figure 3. Relative to individuals’ wearable-measured baseline physiology, wearable-measured physiology changed substantially on the nights before and after self-reported fever days (Figure 3) and exhibited greater deviations in the subset of participants (*n* = 103) with fever days in which self-reported temperatures were greater than 39 °C (red lines, Figure 3). Across all participants with fever days, wearable measured physiology changed the most on the nights before and after fever days (Nights −1 and 0, Figure 3).

**Figure 4:**
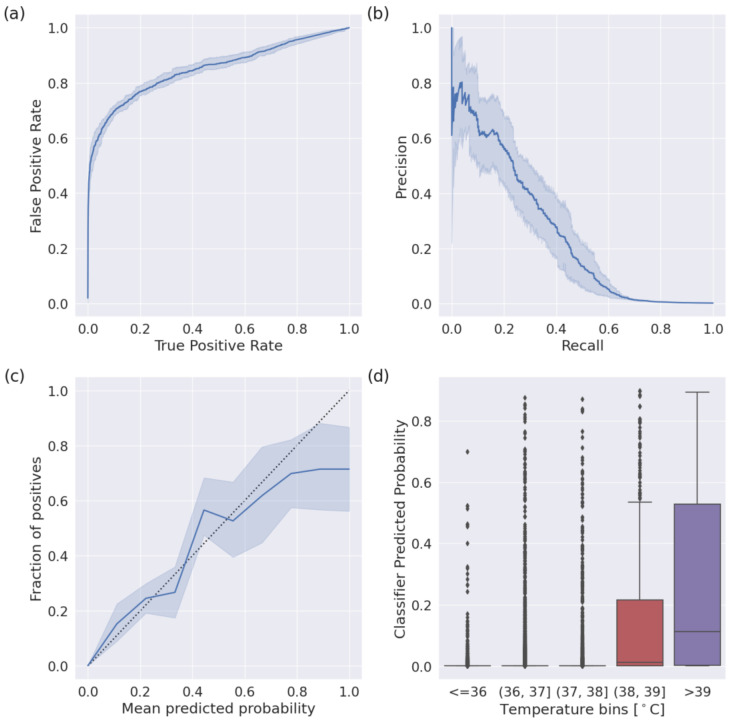
Performance of the fever detection classifier following a five-fold cross-validation scheme. Shaded areas indicate a 95% confidence interval. (**a**) The mean Receiver Operator Characteristic curve (ROC) across iterations. The mean area under the curve is 0.85. (**b**) The mean Precision–Recall curve (PRC) across iterations. The average precision was 0.25. (**c**) The reliability plot (or calibration curve) across iterations. The mean Brier score was 0.0018. (**d**) Box plots indicating the classifier predicted probability, binned by self-reported body temperature.

We depicted model performance following a five-fold cross-validation scheme in Figure 4. The mean AUROC was 0.85 (Figure 4a), and the mean AP was 0.25 (Figure 4b). Our model was well calibrated (Figure 4c) with a Brier score of 0.0018. When considering the aggregated predictions on the test set of each cross-validation, the positive class predicted that probabilities increased with increased self-reported body temperature (Figure 4d) and were significantly correlated (Pearson’s r = 0.11, *p* < 0.001); at a sensitivity of 0.50, the false positive rate was 0.8%.

**Figure 5:**
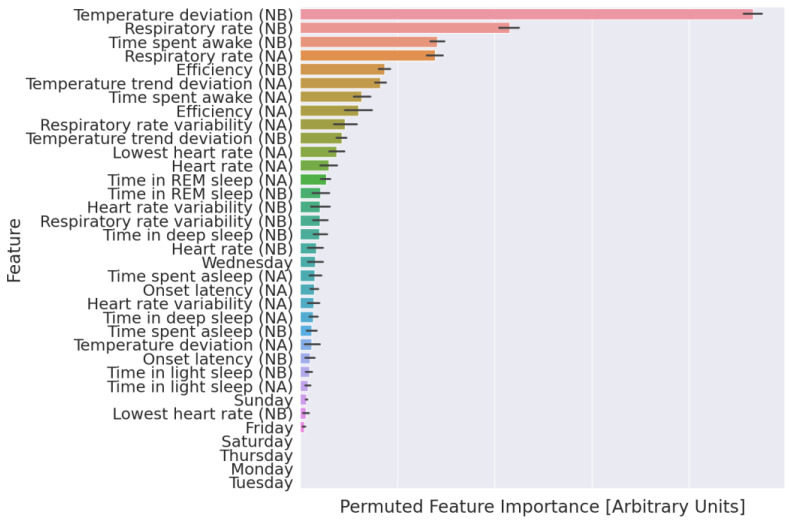
Explanation of the fever detection classifier. Features are ranked from most (top) to least (bottom) important based on the mean permuted importance across 30 permutations. NB: Night before [non]-fever day; NA: night after [non]-fever day; days of the week (i.e., Sunday) indicate the ground truth day; error bars: 95% confidence interval of the mean.

We calculated the permutation importance at each iteration of the five cross-validations. Permutation importance suggested that temperature deviation from the night before a fever day was the most important feature (Figure 5), followed by respiratory rate and the time spent awake the night before the ground truth day.

## 4. Discussion

We found support for the hypothesis that data from wearable devices can be used to detect fevers with high accuracy on the night after the day an individual starts to experience a fever. Specifically, we described wearable measured physiological changes around fever onset (Figure 3) and developed features that were quite computationally tractable and had direct physiological interpretations. Our classifier performed well (average AUROC = 0.85, AP = 0.25) and could be tuned to a sensitivity of 0.50, where it exhibited a false positive rate of 0.8%.

Over a large population, detection using wearable devices could provide important new alerting functionality to SS efforts. Since our model inclusion criteria only required retrievable wearable device data over a two-week baseline period, our model could make predictions on any new device users after about a month of continuous wear time. We calibrated our classifier so that higher predicted examples were more likely to be from a fever day, and our classifier could show promise for a body temperature regression task; the predicted probability increased proportionately to the self-reported body temperature that described a fever. We posit that features with explicit physiological interpretations allow better generalizability to heterogeneous populations than features learned by deep neural networks using a similarly sized training set and believe this to be a key next step following from this work.

Readers should interpret these results in light of our classifier implementation, performance metrics selection, and definition of illness and non-illness periods. While our classifier exhibited sensitive and specific fever onset detection using wearable-measured physiological data in a diverse population, further testing should systematically compare the current classifier implementations across a range of benchmark datasets to determine which classifiers should be further evaluated for deployment. We chose a machine learning architecture that was relatively simple and common to train our classifier; however, there is a wide diversity of approaches used to classify illness from wearable device data (for review, see Mitratza and colleagues) [13]. Furthermore, certain binary classification performance metrics (i.e., AUROC, accuracy) can lead to misleading notions of performance when used on datasets that exhibit extreme class imbalance, as in these analyses where the number of non-fever days far outnumber fever days. Such a class imbalance is common in illness detection studies [27]. Accordingly, we attempted to report all metrics in a way that did not overestimate the performance. A systematic comparison of illness detection classifiers would require consistent definitions of illness and non-illness periods across benchmark datasets, as well as the use of the same metrics to describe classifier performance across these datasets.

This work also differs from other illness detection studies in both study design and the wearable device used to gather data. We performed these analyses retrospectively, and the performance should be verified in a prospective manner [27]. Furthermore, differences in commercially available wearable device sensors (i.e., the ability to collect HRV, HR, temperature, and other physiological metrics) have led to substantial differences in the features used to train illness detection classifiers. We trained our classifier using data from second-generation Oura Rings, which, at the time of data collection, were different from most other wearable devices in that they included a temperature sensor, which was not included in most other wearable devices of similar cost and market penetration (i.e., Apple Watch and FitBit). Regardless of feature differences, data from wearable devices without temperature sensors have been used to train many of the other previously studied illness detection classifiers over the past decade [7,15]. However, many of the most recent generations of wearable devices from Apple, FitBit, and Whoop now include a temperature sensor. Future work should investigate if and how different sensors in wearable devices create features that improve illness detection performance, particularly because our results suggest that temperature sensor-based features are the most important in our classifier (Figure 5). Measurements from sensors not traditionally included in commercial wearable devices, such as those that monitor analytes in sweat [34] or exhaled air [35], might be particularly important for improving the accuracy of these models. Other efforts have engineered more complicated features, i.e., features based on deviations from expected circadian rhythms [36]; here, we demonstrate an impressive performance using nightly summary data. Researchers should systematically explore the effects of the study design and wearable device features as they work toward developing standards of real-world efficacy.

Our specific algorithmic implementation requires a minimum level of wearable device compliance. Previous work based on the dataset we used here demonstrates that participants exhibit a high level of wearable device compliance (87.8% of nights) [37]. Another survey-based study found that 72.58% of participants in their study wore their wearable device “daily” or “almost daily” [5]. Future work could weigh certain metrics like recall against the proportion of days wherein users provide enough data to produce variable results in order to determine the efficacy of these models.

As with other health-screening applications, illness detection algorithms based on wearable device data need to balance improving case detection with minimizing false positives. Illness detection generalizability should also be carefully evaluated across classifier implementations, the wearable devices used, and diverse populations. In particular, researchers should address whether models generalize across geographic regions. Future work should also examine whether the performance of illness detection models varies temporally. Such temporal performance variability might be driven by seasonality in illness prevalences. Once models exhibit a performance that can have a real-world impact, developments in wearable device data deidentification and data integration at public health agencies will be crucial to developing systems for real-time illness monitoring. Data privacy and deidentification are challenges that remain largely unaddressed for wearable device data. Recent works further demonstrate how it might be possible to re-identify individuals using de-identified wearable device data [38]. Furthermore, as of 2024, these data fall under the category of “personal health data” in the EU [39] and US [40], and these data are subject to regulations that vary by jurisdiction. However, it is possible that the categorization of these data might change in the future, along with the regulations they are subject to. Finally, our efforts suggest that symptom screening classifiers that generalize across illnesses may be a useful public health tool for real-time surveillance.

## Figures and Tables

**Table 1:** Detailed descriptions of each wearable measured sleep summary feature.

Metric	Unit of Measurement	Description
Heart rate	Beats per minute	The average heart rate registered during the sleep period.
Lowest heart rate	Beats per minute	The lowest heart rate (5 min sliding average) registered during the sleep period.
Heart rate variability	Milliseconds	The average HRV calculated using the rMSSD method.
Respiratory rate	Breaths per minute	Average respiratory rate.
Respiratory rate variability	Breaths per minute	The average variability of respiratory rate (STD) in the sleep period.
Temperature deviation	Degrees Celsius	Skin temperature deviation from the user’s long-term temperature average.
Temperature trend deviation	Degrees Celsius	Skin temperature deviation from weighted three-day rolling temperature average.
Onset latency	Seconds	Detected latency from the time the user entered their bed to the beginning of the first five minutes of persistent sleep.
Time spent awake	Seconds	Total amount of awake time registered during the sleep period.
Time spent in REM sleep	Seconds	Total amount of REM sleep registered during the sleep period.
Time spent in light sleep	Seconds	Total amount of light (N1 or N2) sleep registered during the sleep period.
Time spent in deep sleep	Seconds	Total amount of deep (N3) sleep registered during the sleep period.
Time spent asleep	Seconds	Total amount of sleep registered during the sleep period.

**Table 2:** The number of individuals included in the training and test sets, including self-reported sex assigned at birth, age, and race.

		Dataset Composition
N		16,794
Sex, n (%)	Female	7324 (43.6)
	Male	9455 (56.3)
	Other	15 (0.1)
Age, mean (SD)		47.2 (12.3)
Race, n (%)	African American/Black	226 (1.4)
	East Asian	685 (4.2)
	Caucasian/White	14,120 (86.3)
	Middle Eastern	94 (0.6)
	Native American/Native Alaskan	27 (0.2)
	Native Hawaiian or Other Pacific Islander	28 (0.2)
	South Asian	162 (1.0)
	Other	429 (2.6)
	Prefer not to answer	596 (3.6)

## Data Availability

Oura’s data use policy does not permit us to make the data available to third parties. Therefore, those seeking to reproduce the findings in this manuscript should contact the corresponding author P.K. The distribution of the source code is limited by the Department of Defense and, therefore, it cannot be shared.

## Appendix A

### Appendix A.1. Oura Ring Gen2 Sensor Specifications and Algorithm Descriptions

Many of the technical sensor specifications, signal-processing steps, and algorithms that the Oura Ring Gen2 uses to calculate physiological metrics are proprietary; however, certain details are publicly available. We summarize those details here.

#### Appendix A.1.1. Sensors

Temperature: The Oura Ring measures temperature with two negative temperature coefficient (NTC) thermistors (non-calibrated, resolution of 0.07 degrees Celsius) located palmar when the ring is worn as intended. Accelerometry: the Oura Ring includes a 50 Hz, ±2 g triaxial accelerometer [26]. Photoplesmogram: the photoplethysmogram consists of a photodetector that, when the ring is worn as intended, is positioned in the palmar middle of the finger with two 900 nm LEDs on either side of the photodetector. Raw photoplethysmography (PPG) is sampled at 250 Hz [26,41].

##### Appendix A.1.2. Heart Rate, Heart Rate Variability, and Respiratory Rate

The Oura Ring calculates heart rate (HR), heart rate variability (HRV), and respiratory rate (RR) from inter-beat intervals (IBIs) during periods of sleep. IBIs are calculated using raw PPG data processed using a real-time moving average filter [26,41]. The local maximum and minimum values in these PPG data correspond to each heartbeat. The Oura Ring also estimates the probability that each IBI is an artifact. The Oura Ring uses a median filter to classify each IBI as either normal or abnormal; IBIs more than 16 bpm removed from the median IBI in a moving window of length seven are marked as abnormal [26,41]. If any of the two IBIs before or after a particular IBI are abnormal, that IBI is marked as abnormal. HR and HRV are calculated if at least 30% of the IBIs in a 5 min window are normal according to these criteria [26,41]. The Oura Ring calculates HR using the mean IBI and HRV as the root mean square of successive differences (rMSSD). RR is calculated by finding peaks in IBI over the time period under analysis [26,41]. These metrics are generated on-device, and the raw PPG is not continuously recorded or stored for analysis.

#### Appendix A.1.3. Sleep Stages

The Oura Ring calculates sleep stages using a machine-learning classifier and predicts sleep stages on 30 s windows of data [26]. The Oura Ring assesses temperature at 10 s intervals and samples less than 31 or more than 40 degrees Celsius are masked [26]. The mean, min, max, and standard deviation are calculated on a rolling basis [26]. High frequency accelerometry data are used to calculate the mean amplitude deviation (MAD) in 5 s windows [26]. The MAD represents the average deviation from the mean vector magnitude [26]. Within each 30 s window, the mean, max, and interquartile range (IQR) of MADs in the 10–90th percentile of the window are calculated [26]. The difference in arm angle was also calculated in each 5 s window and the mean, max, and IQR of arm angles in the 10–90th percentile of the 30 s window are calculated [26]. Processed accelerometry features are also calculated for each three individual axes [26]. High-resolution data are processed using a 5th-order Butterworth bandpass filter (3 to 11 Hz) and taking their absolute value [26]. The mean, max, and IQR of values in the 10–90th percentile within each axis are calculated for each 30 s window [26]. High-quality IBIs are identified in the same way they are identified for the calculation of HR, HRV, and RR in 5 min windows [26]. For each 30 s window, Oura calculates HR, HRV (rMSSD), and RR [26]. They also calculate the following additional HRV metrics: SDNN, pNN50, frequency power in the LF and HF bands, the main frequency peak in the LF and HF bands, total power, normalized power, mean and coefficient of variation in the zero-crossing interval. Excluding accelerometer-based features, each feature is normalized on a per-night basis using the 5–95 percentiles of that feature [26]. Oura claims this accounts for inter-individual differences in features.
